# Lifestyle and psychosocial factors associated with maintenance of normal body mass index in college students: a cross sectional study

**DOI:** 10.1186/s13104-020-05362-1

**Published:** 2020-11-10

**Authors:** Bengt B. Arnetz, Thomas N. Templin, K.-L. Catherine Jen, Sukhesh Sudan, Judith E. Arnetz

**Affiliations:** 1 Department of Family Medicine, College of Human Medicine, Michigan State University, 15 Michigan Street NE, Grand Rapids, MI 49503 USA; 2 College of Nursing, Wayne State University, Detroit, MI USA; 3 Department of Nutrition and Food Science, Wayne State University, Detroit, MI USA

**Keywords:** Weight maintenance, Body mass index, Psychosocial health, University, Academic year

## Abstract

**Objectives:**

The purpose of this study was to identify lifestyle and psychosocial factors associated with maintenance of normal body mass index (BMI, 18.5–24.9 kg/m^2^). Undergraduate students (n = 2781; 7.1% response rate) at a Big Ten university responded to a survey in 2018. BMI was calculated from the reported weight and height at the time of the survey and upon entering the university. Logistic regression analyses examined lifestyle and psychosocial health factors associated with maintenance of normal BMI by academic year.

**Results:**

Current BMI was within normal range for 68.8% of freshmen and 60.6% of seniors. Never consuming fast food was a significant predictor for maintaining normal BMI in sophomores (OR 3.78; 95% CI 1.61, 8.88; *p* < 0.01) and juniors (OR 7.82; 95% CI 2.14, 28.65; *p* < 0.01). In seniors, better psychosocial health (OR 1.40; 95% CI 1.12, 1.76; *p* < 0.01) was the only significant predictor for maintaining one’s normal freshman BMI category. Among those within the normal BMI range upon entering the university, psychosocial health (OR 1.31; 95% CI 1.10, 1.55; *p* < 0.01) was the only predictor of retaining one’s absolute BMI within ± 3% as a senior. Prospective studies are needed to better understand the interaction between environment, behavior, and psychological health involved in retaining normal weight.

## Introduction

The World Health Organization (WHO) lists overweight and obesity as serious health issues facing both the developed and developing world [1, 2]. A literature review revealed that for every 1000 articles that focused on overweight and obesity, fewer than 5 articles addressed maintenance of normal weight (search criteria available upon request). Weight homeostasis, the ability to maintain a normal body mass index (BMI between 18.5–24.9 kg/m^2^), is an indicator of integrated wellness. Our theoretical model is informed by the stress paradigm and the concepts of homeostasis vs allostasis in terms of the organism’s ability to maintain an internal steady state, e.g., keeping blood glucose within a normal range, despite a constant change in the external environment [3, 4]. Undergraduate college students are an excellent group to study since this is a period in life where overweight and obesity typically develop [5–7]. Moreover, they are exposed to a rather homogenous environment that can either be obesogenic or salutogenic, depending on environmental cues, personal characteristics, and lifestyle choices [8, 9]. Students are exposed to both eustress (“beneficial” stress) and distress which differentially impact the limbic system that is critical in regulating emotions, behavior, and caloric intake [10, 11]. This study explored demographic, lifestyle and psychosocial health factors associated with maintaining one’s normal BMI from the time of starting the university to the time of the survey.

## Main text

### Methods

#### Setting and study participants

This study was conducted in September of 2018 at Michigan State University (East Lansing, Michigan) that has universally applied requirements and benefits. This allowed us to account for confounders otherwise difficult to control for in weight studies. All freshmen are required to live on campus and participate in a meal plan, contributing to “unlimited” access to food and non-alcoholic beverages. Students also have free access to a broad array of weight protective factors, e.g., gyms, cycle paths, and other outdoor recreational environments.

All undergraduate students (N = 39,423) were invited to participate in the study. A questionnaire was distributed to the students’ official university email address using a secure, online survey system (Qualtrics, Provo, UT). The email included detailed information about the study and its purpose with information as to how students could get additional information. No participant incentives were provided. A total of 3204 (response rate = 8.1%) students clicked on the link and agreed to participate in the study. However, 423 students did not respond to any question and the final sample consisted of 2781 students (response rate = 7.1%). The study group differed significantly from the entire pool of university undergraduates in terms of gender (68% females in study group vs. 51% at the university), race (83% Whites in study group vs. 68%) and international students (5.9 vs. 10.2%).

#### Survey

A 24-item survey covered demographics, current weight and height, weight change since entering the university, psychosocial health [12, 13], exercise [14], eating/drinking habits, and consumption of tobacco products [15]. Demographic variables included academic year (Freshman, Sophomore, Junior or Senior), sex, race/ethnicity, and country of birth.

##### Body mass index and perceived weight change

Reported height in inches and weight in pounds was used to calculate *current* BMI in kg/m^2^ using the Center for Disease Control and Prevention (CDC) formula by dividing weight in pounds (lbs) by height in inches (in) squared and multiplying by a conversion factor of 703 [16]. Retrospective BMI was based on participants’ response to “How has your weight changed since you started at the university?” with the response options, increased, remained same or decreased. Those who reported that their weight increased or decreased responded to a follow up question on how much weight (in lbs) they had gained or lost, respectively. Participants’ height was assumed to remain stable throughout university. Participants were classified into underweight, normal, overweight and obese based on CDC BMI categories [16] both at the time of the survey and when entering the university. Maintenance of the *absolute BMI* in study participants was defined as remaining within ± 3% of their retrospective BMI [17].

##### Psychosocial health and exercise

Psychosocial health was calculated as the sum of the participants’ ratings of energy [12], health [13], satisfaction with social life, and stress (reverse scoring) [12] on validated visual analog scales (VAS) ranging from 1–10 (Additional file 1). The participants’ response to questions “How is your energy level right now”, “How is your health right now”, “Are you satisfied with your social life right now” and “How stressed are you right now” were used to assess their energy, health, satisfaction with social life and stress, respectively. The sum score was divided by 4 yielding an index ranging from 1 (low) to 10 (high). Higher scores denote better psychosocial health. The participants responded to a VAS question, “How often do you exercise” on a scale of 1 (never) to 10 (daily) [9].

##### Dietary habits

Participants reported the number of daily meals they consumed. Responses were dichotomized into—less than 3 meals and 3 or more meals. Reported consumption of fast food, sugar-sweetened, and alcoholic beverages were each dichotomized into—Never versus at least once per week.

##### Tobacco

Consumption of cigarettes, cigars, chewing tobacco/snuff, e-cigarettes and electronic vaping products was reported on a yes/no response [15].

#### Data analysis

Statistical analysis was conducted using IBM SPSS statistics, V.25, 2018 (IBM Corp, Armonk, NY). Chi-square analysis was used to test for significant differences by academic year in demographics, dietary habits, tobacco consumption, current and retrospective *BMI categories*, and weight change since entering the university. One-way ANOVA was used to test for significant differences by academic year in exercise, *absolute BMI* at the time of entering the university and at the time of the survey, and psychosocial health. Separate multivariable logistic regressions for sophomores, juniors and seniors were used to identify factors associated with maintaining normal *BMI category* from freshman year. Demographics, exercise, dietary habits, tobacco consumption and psychosocial health ratings were used as independent variables. Separate multivariable logistic regressions were used with the same independent variables to identify factors associated with seniors maintaining their normal *absolute BMI* (defined as BMI remaining within ± 3%) [17] from the time they entered the university.

Since there were missing cases on some of the variables (ranging from 0.1 to 10%), we ran regression analyses with and without multiple imputation [18]. Results were similar and only non-imputed results are reported. Statistical significance was set to a two-sided p-value of < 0.05.

## Results

Characteristics of all survey respondents by academic year are shown in Table 1. A larger proportion was female, White, U.S. born and non-tobacco users. The participants did not differ by academic year based on gender, U.S. born, number of meals consumed per day, consumption of tobacco products, and exercise. The proportion of Whites increased from freshman to senior year while that of Asians and Blacks decreased (*p* = 0.02). The proportion that consumed fast food (*p* < 0.001) or alcoholic beverage (*p* < 0.001) at least once per week was higher in both juniors and seniors as compared to freshmen and sophomores. In contrast, sweetened beverage consumption at least once per week was lower in juniors and seniors as compared to freshmen and sophomores (*p* < 0.001).

**Table 1:** Characteristics of survey respondents by academic year

	All (n = 2781)	Freshmen (n = 860)	Sophomore (n = 636)	Junior (n = 577)	Senior (n = 694)	*P* value^4^
	n (%)	n (%)	n (%)	n (%)	n (%)	
Gender						0.17
Male	855 (30.9)	274 (31.9)	168 (26.4)	192 (33.3)	220 (31.7)	
Female	1883 (68.0)	577 (67.2)	459 (72.2)	380 (65.9)	465 (67.0)	
Other^5^	31 (1.1)	8 (0.9)	9 (1.4)	5 (0.9)	9 (1.3)	
US born						0.13
Yes	2602 (94.1)	811 (94.7)	604 (95.1)	529 (92.2)	655 (94.0)	
No	163 (5.9)	45 (5.3)	31 (4.9)	45 (7.8)	42 (6.0)	
Race						0.02
White	2212 (79.5)	656 (76.3)	506 (79.6)	472 (81.7)	576 (82.6)	
Black/African American	107 (3.8)	41 (4.8)	23 (3.6)	18 (3.1)	25 (3.6)	
Asians	201 (7.2)	80 (9.3)	54 (8.5)	30 (5.2)	36 (5.2)	
Other	261 (9.4)	83 (9.7)	53 (8.3)	58 (10.0)	60 (8.6)	
No. of meals per day						0.63
Less than 3 meals	1400 (50.4)	428 (49.8)	322 (50.7)	302 (52.4)	341 (48.9)	
3 or more meals	1378 (49.6)	432 (50.2)	313 (49.3)	274 (47.6)	356 (51.1)	
Fast food consumption per week						< 0.001
At least once	1550 (55.7)	407 (47.3)	324 (50.9)	357 (61.8)	456 (65.4)	
Never	1231 (44.3)	453 (52.7)	312 (49.1)	221 (38.2)	241 (34.6)	
Sweetened beverages consumption						< 0.001
At least once/week	2126 (76.4)	699 (81.3)	495 (77.8)	420 (72.7)	505 (72.5)	
Never	655 (23.6)	161 (18.7)	141 (22.2)	158 (27.3)	192 (27.5)	
Alcoholic beverages consumption						< 0.001
At least once/week	1655 (59.5)	385 (44.8)	365 (57.4)	362 (62.6)	536 (76.9)	
Never	1126 (40.5)	475 (55.2)	271 (42.6)	216 (37.4)	161 (23.1)	
Tobacco products consumption						0.06
Yes	597 (21.5)	207 (24.2)	129 (20.3)	127 (22.0)	130 (18.7)	
No	2179 (78.5)	650 (75.8)	507 (79.7)	451 (78.0)	565 (81.3)	
Reported retrospective weight status upon starting the university						0.09
Normal weight^1^	1734 (65.7)	550 (68.5)	387 (65.0)	346 (62.3)	446 (65.9)	
Overweight/Obese^1^	753 (28.5)	208 (25.9)	181 (30.4)	177 (31.9)	183 (27)	
Underweight^1^	152 (5.8)	45 (5.6)	27 (4.5)	32 (5.8)	48 (7.1)	
Current weight status						0.03
Normal weight	1738 (64.1)	575 (68.8)	389 (63.3)	354 (62.5)	415 (60.6)	
Overweight/Obese	842 (31.0)	218 (26.1)	198 (32.2)	187 (33.0)	234 (34.2)	
Underweight	132 (4.9)	43 (5.1)	28 (4.6)	25 (4.4)	36 (5.3)	
Perceived weight change since starting at the university						< 0.001
I ncreased	947 (35.1)	187 (22.6)	198 (32.3)	204 (36.1)	358 (52.0)	
Remained same	1126 (41.8)	452 (54.5)	246 (40.1)	237 (41.9)	191 (27.8)	
Decreased	622 (23.1)	190 (22.9)	169 (27.6)	124 (21.9)	139 (20.2)	

	Mean (SD)	Mean (SD)	Mean (SD)	Mean (SD)	Mean (SD)	
Exercise (1–10; Never—daily)	5.3 (2.7)	5.4 (2.6)	5.3 (2.7)	5.3 (2.7)	5.4 (2.6)	0.81
Psychosocial well-being (1–10; low–high)	5.3 (1.4)	5.5 (1.4)	5.2 (1.5)	5.2 (1.5)	5.4 (1.4)	0.01
Retrospective BMI^2^	23.8 (5.1)	–	24.1 (5.1)	24.0 (5.2)	23.6 (5.2)	0.14
Current BMI^3^	24.1 (5.1)	23.6 (4.9)	24.1 (5.0)	24.3 (5.1)	24.5 (5.4)	0.01

^1^Categorized based on CDC classification guidelines Available at: https://www.cdc.gov/healthyweight/assessing/bmi/index.html; ^2^Retrospective BMI was computed from the participants’ reports of their weight when entering the university; ^3^Current BMI was computed from self-reported height and weight at the time of the study; ^4^represents significant differences across academic years using chi-square for categorical and one-way ANOVA for continuous variables; ^5^included students that identified as “other” gender

Current *BMI category* was significantly related to academic year: 68.8% of freshmen reported being in the normal category at the time of the survey as compared to 60.6% of the seniors (*p* = 0.03; Table 1). Participants’ reported *absolute BMI* at the time of starting at the university did not differ by academic year (*p* = 0.14). However, reported weight change from the time of entering the university until the time of the survey was related to current academic year, with 27.8% of seniors reporting that their weight remained the same as their freshmen weight, and 52.0% reporting weight gain (*p* < 0.001). Current *absolute BMI* increased across the academic years from 23.6 for freshmen to 24.5 for seniors (*p* = 0.01). Approximately one third (35.1%) of seniors within the normal *BMI category* when starting at the university remained within ± 3% of freshmen *absolute BMI* (Data not shown).

Table 2 depicts results for multivariable logistic regressions for factors associated with remaining within the normal *BMI category* since entering the university. Sophomores (OR 3.78; 95% CI 1.61, 8.88; *p* < 0.01) and juniors (OR 7.82; 95% CI 2.14, 28.65; *p* < 0.01) that never consumed fast food were more likely to remain within the normal *BMI category*. Seniors with higher ratings on psychosocial health (OR 1.40; 95% CI 1.12, 1.76; *p* < 0.01) were more likely to report having remained in the normal *BMI category*.

**Table 2:** Multivariable logistic regression for factors associated with remaining within the normal^1^ BMI category from freshman year until responding to the current survey (n = 995)

	Sophomore; (n = 323)	Juniors (n = 284)	Seniors (n = 388)
	OR^2^ (95% CI^3^)	OR (95% CI)	OR (95% CI)
Demographics			
Females (Ref. Males)^4^	0.64 (0.24, 1.73)	1.77 (0.70, 4.49)	1.07 (0.56, 2.05)
Race (Ref. White)			
Black	0.89 (0.16, 4.92)	–^5^	–^5^
Asians	1.61 (0.42, 6.21)	1.35 (0.14, 13.44)	3.31 (0.37, 29.97)
Other	0.67 (0.18, 2.54)	0.66 (0.18, 2.50)	0.67 (0.25, 1.80)
Born outside US (Ref. US born)	1.67 (0.18, 15.18)	1.80 (0.30, 10.73)	2.70 (0.30, 24.54)
Exercise; 1 (never)— 10 (daily)	1.05 (0.90, 1.24)	0.97 (0.82, 1.15)	1.07 (0.96, 1.21)
Dietary habits			
Consumed ≥ 3 meals/day (Ref. < 3 meals/day)	0.77 (0.34, 1.76)	0.58 (0.24, 1.40)	1.70 (0.95, 3.06)
Never consumed fast food (Ref. at least once/week)	3.78 (1.61, 8.88)^**^	7.82 (2.14, 28.65)^**^	0.91 (0.48, 1.72)
Never consumed beverages (Ref. at least once/week)	1.55 (0.55, 4.33)	0.55 (0.21, 1.41)	1.52 (0.77, 3.00)
Never consumed alcohol (Ref. at least once/week)	0.54 (0.24, 1.20)	0.64 (0.25, 1.64)	0.54 (0.26, 1.11)
Tobacco consumption			
No (Ref. Yes)	0.92 (0.30, 2.81)	1.31 (0.46, 3.73)	1.26 (0.62, 2.56)
Psychosocial health; 1 (low)—10 (high)	0.99 (0.75, 1.31)	0.96 (0.70, 1.30)	1.40 (1.12, 1.76)^**^
Nagelkerke R^2^	0.10	0.15	0.14

**p < 0.01; ^1^Normal BMI as defined by CDC categorization; ^2^Odds ratio; ^3^95% confidence interval; ^4^Other category was excluded; ^5^Statistics could not be computed as there were too few individuals; n is smaller than the number who responded as some cases were excluded from the regression analysis due to missing values for some variables

Table 3 shows multivariable logistic regression for factors associated with weight homeostasis in seniors that as freshman were classified within the normal *BMI range* and did not deviate more than ± 3% from their freshman *absolute BMI* until the current survey. Better psychosocial health (OR 1.31; 95% CI 1.10, 1.55; *p* < 0.01) was the only significant predictor.

**Table 3:** Multivariable logistic regression for factors associated with seniors that remained within their normal BMI^1^ range as freshmen based on deviating less than ± 3% in their absolute BMI (n = 388)

	OR^2^ (95% CI^3^)
Demographics	
Females (Ref. Males)^4^	0.99 (0.61, 1.61)
Race (Ref. White)	
Black	–^5^
Asians	2.11 (0.77, 5.81)
Other	0.66 (0.28, 1.58)
Born outside US (Ref. US born)	1.20 (0.41, 3.52)
Exercise; 1 (never)—10 (daily)	0.99 (0.91, 1.08)
Dietary habits	
Consumed ≥ 3 meals/day (Ref. < 3 meals/day)	0.98 (0.62, 1.54)
Never consumed fast food (Ref. at least once/week)	0.93 (0.58, 1.50)
Never consumed beverages (Ref. at least once/week)	1.10 (0.69, 1.78)
Never consumed alcoholic beverages (Ref. at least once/week)	0.90 (0.49, 1.64)
Tobacco consumption	
No (Ref. Yes)	0.97 (0.54, 1.74)
Psychosocial wellbeing; 1 (low)—10 (high)	1.31 (1.10, 1.55)**
Nagelkerke R^2^	0.07

**p < 0.01; ^1^Normal BMI category according to CDC criteria; ^2^Odds ratio; ^3^95% confidence interval; ^4^Other category was excluded; ^5^Statistics could not be computed as there were too few individuals

### Discussion

The current study contributes to limited research focusing on factors associated with remaining within the normal BMI category during the emerging adult years while attending a university. With advancing academic years, the importance of psychosocial health increased. We posit that psychosocial factors are an “upstream” regulator of lifestyle “choices” [19, 20]. Thus, if a person is in good psychosocial health, there is no need for the brain to prepare the flight or fight response, which requires energy mobilization achieved by changes in neuroendocrine secretion patterns [10]. A person under chronic stress is more likely to exhibit pathophysiological changes contributing to metabolic syndrome and insulin resistance—conditions closely affiliated with weight increases [10, 11, 21]. In contrast, eustress or beneficial stress has been purported to be associated with higher ratings on wellbeing and may be indicative of better psychosocial health which may lead to students making better health choices and thereby supporting weight maintenance [22].

Age-related weight increase in university students has been reported in several studies although as a group college graduates are usually at reduced risk of becoming overweight or obese compared to those with shorter education [5–7]. However, there are only a few prior studies addressing weight maintenance in a semi-controlled environment, such as a university [23, 24].

Psychosocial health was the only significant predictor in seniors that reported that they had been able to maintain their normal BMI since entering the university. Sustained psychosocial stress is known to result in altered eating patterns, pursuit of energy dense food, and, commonly, decrease in exercise [10, 25]. In the current study, psychosocial health, that is, the opposite to psychosocial stress, was independently associated with seniors remaining both within the normal *BMI category* and deviating less than ± 3% from the original normal *absolute BMI* as freshman. This held true even after accounting for consumption of fast food and exercise. A prospective study followed African American adults with a baseline BMI ≥ 18.5 kg/m^2^ for a period of 5 years [26]. Important determinants of maintaining one’s weight or losing vs. gaining weight were healthy nutritional habits and not residing in an unsafe neighborhood which is indicative of decreased psychosocial health.

Future studies are needed to longitudinally investigate the inter-relationship between personal, biological, lifestyle, and environmental factors and the likelihood of maintaining normal BMI during college. This is a period with substantial personal and intellectual growth that, at the same time, can be highly emotionally stressful [25]. Finally, studies of undergraduates might offer us a novel window into determinants of the establishment of lifestyle, behavioral and cognitive coping patterns that might be lifelong, and how these relate to maintenance of normal weight [24].

## Limitations

All data were based on self-reports. The participants’ reported height at the time of the study was used to compute their retrospective BMI. The study was cross-sectional which limits our ability to separate possible age cohort from academic year effects. The study was conducted at a single university and the response rate, although not unusually low for these kinds of studies, represents less than 10% of eligible respondents [27].

## Supplementary information

**Additional file 1.** Survey instrument.

## Data Availability

The data sets used and/or analyzed during the current study are available from the corresponding author upon request.

## Abbreviations

WHO: World Health Organization
BMI: Body mass index
CDC: Center for Disease Control and Prevention
VAS: Visual analog scale
