# Supplementary material for: Lifestyle and psychosocial factors associated with maintenance of normal body mass index in college students: a cross sectional study
Source: BMC Res Notes. 2020 Nov 10;13:516. doi: 10.1186/s13104-020-05362-1 (PMC7653865; doi:10.1186/s13104-020-05362-1)
Supplement: Supplementary file 1 — Additional file 1. Survey instrument. [file 13104_2020_5362_MOESM1_ESM.docx]

**Student Survey (Qualtrics)**

**Stress and Energy**

1. How is your health right now?

1 3 5 8 10

Average

Very Poor

Very Good

1. How did you sleep last night?

1 3 5 8 10

Average

Very Good

Very Poor

1. How is your ability to concentrate right now?

1 3 5 8 10

Average

Very Good

Very Poor

1. How stressed are you right now?

1 3 5 8 10

Very stressed

Not at all

Average

1. How is your energy level right now?

1 3 5 8 10

No energy

Average

Full of energy

1. Are you satisfied with your social life right now?

1 3 5 8 10

Average

Very satisfied

Very dissatisfied

1. How often do you exercise?

1 3 5 8 10

Never

Every day

Average

**Eating Habits**

1. How many meals do you typically eat/day:

❒ 1 meal/day ❒ 2 meals/day ❒ 3 meals/day ❒ 4 meals/day ❒ 5 or more meals/day

1. Do you prepare your own meals:

❒ Every day ❒ 5-6 days/week ❒ 3-4 days/week ❒ 1-2 days/week ❒ Never

1. How often do you eat fast food:

❒ Every day ❒ 5-6 days/week ❒ 3-4 days/week ❒ 1-2 days/week ❒ Never

1. How often do you drink sugar-sweetened or diet beverages (soda; flavored juice drinks,

sports drinks, sweetened tea, coffee drinks, energy drinks, etc.):

❒ Every day ❒ 5-6 days/week ❒ 3-4 days/week ❒ 1-2 days/week ❒ Never

1. How often do you drink alcohol beverages (beer; wine; liquor; cocktail, etc.):

❒ Every day ❒ 5-6 days/week ❒ 3-4 days/week ❒ 1-2 days/week ❒ Never

**Tobacco/Nicotine Products**

1. Do you:

Smoke cigarettes: ❒ Yes ❒ No

Smoke cigars ❒ Yes ❒ No

Use chewing tobacco/snuff: ❒ Yes ❒ No

Use E-cigarettes: ❒ Yes ❒ No

Use electronic vaping products (e.g. e-hookahs) ❒ Yes ❒ No

**Biometric Information**

1. What is your height (inches or centimeters): __________
2. What is your weight (pounds or kilograms): __________
3. How has your weight changed since you started at MSU:

❒ Increased, if so how much (lb or kg): __________

❒ The same

❒ Decreased, if so how much (lb or kg): __________

**Demographic Information**

1. Are you a: ❒ Freshman ❒ Sophomore ❒ Junior ❒ Senior
2. Do you live in college housing (e.g. MSU residence hall)? ❒ Yes ❒ No
3. Is your major at MSU health related (e.g. pre-med, biology, etc.) ❒ Yes ❒ No
4. What is your gender? ❒ Male ❒ Female ❒ Other
5. Were you born in the United States: ❒ Yes ❒ No
6. Did you come to the United States for your undergraduate studies: ❒ Yes ❒ No ❒ N/A
7. What is your race (drop down list):

❒ White

❒ Black or African American

❒ American Indian or Alaska Native

❒ Asian Indian

❒ Chinese

❒ Filipino

❒ Japanese

❒ Korean

❒ Vietnamese

❒ Native Hawaiian or Other Pacific Islander

❒ Other: __________________________________________

1. Are you Hispanic or Latino? ❒ Yes ❒ No

**Thank you for taking the time to complete this *confidential* survey!**
